# Clinical Outcomes of Shunting in Normal Pressure Hydrocephalus: A Multicenter Prospective Observational Study

**DOI:** 10.3390/jcm11051286

**Published:** 2022-02-26

**Authors:** Sokol Trungu, Antonio Scollato, Luca Ricciardi, Stefano Forcato, Filippo Maria Polli, Massimo Miscusi, Antonino Raco

**Affiliations:** 1 Neurosurgery Unit, Card. G. Panico Hospital, 73039 Tricase, Italy; ascollat@hotmail.com (A.S.); stefano.forcato@gmail.com (S.F.); 2 Department of Neuroscience, Mental Health and Sense Organs (NESMOS), Sant’Andrea Hospital, Sapienza University of Rome, 00189 Rome, Italy; ricciardi.lu@gmail.com (L.R.); massimo.miscusi@gmail.com (M.M.); antonino.raco@gmail.com (A.R.); 3 Neurosurgery Unit, Catholic University of Rome, 00100 Rome, Italy; filippomaria.polli@gmail.com

**Keywords:** normal pressure hydrocephalus, hydrocephalus, ventriculoperitoneal shunt, NPH, cerebrospinal fluid, VPS, dementia, neurodegenerative diseases

## Abstract

**Background**: Normal pressure hydrocephalus (NPH) is characterized by the triad of dementia, gait disturbance and urinary incontinence, all potentially reversible following a ventriculoperitoneal shunt (VPS). This study aims to evaluate the clinical outcomes of shunting in normal pressure hydrocephalus following a new standardized protocol. **Methods**: This study is designed according to the STROBE guidelines. Demographical, clinical, surgical and radiological data were collected from May 2015 to November 2019. Gait, balance and incontinence data based on the NPH European scale were collected before and after one, six and twelve months of treatment with a VPS. Clinical symptoms and changes of the stoke volume, measured on phase-contrast MRI, were used to evaluate improvement after VPS surgery. **Results**: One hundred and eighty-one consecutive patients met the inclusion criteria. The mean age was 73.1 years (59–86) and mean follow-up was 38.3 months (13–50). The gait (58.5 ± 14.3 to 70.1 ± 13.4, *p* < 0.001), the balance (66.7 ± 21.5 to 71.7 ± 22.1, *p* = 0.001), continence domain (69.9 ± 20.5 to 76 ± 20, *p* = 0.002) scores and neuropsychological scales showed a statistically significant improvement over the follow-up. The overall improvement after 12 months was present in 91.2% of patients. An overall complication rate of 8.8% and a reoperation rate of 9.4% were recorded, respectively. **Conclusions**: Surgical treatment by VPS for NPH improves symptoms in most patients, when accurately selected. A standardized protocol and a multidisciplinary team dedicated to this disorder is needed to achieve an early and correct diagnosis of NPH. Follow-up with stroke volume measurement is a valuable tool for the early diagnosis of shunt malfunction or the need for valve adjustment.

## 1. Introduction

Normal pressure hydrocephalus (NPH) is typically characterized by the triad of dementia, gait disturbance and urinary incontinence, all potentially reversible following a ventriculoperitoneal shunt (VPS). Nevertheless, difficulties in differential diagnosis with neurodegenerative diseases result in a delay and/or lack of treatment, thus preventing recovery with dramatic consequences in terms of clinical deterioration and growing socio-economic costs [1,2].

Pathophysiology of NPH is still debated. Despite the mechanical nature suggested by the name, recent studies focused on periventricular white matter and its vascular supply degeneration as a possible cause [3,4]. NPH diagnosis is based on clinical and radiological exams, which, however, show high variability in terms of sensitivity and specificity, probably due to the heterogeneity of the methods employed [5,6].

However, the diagnosis of NPH is complicated by the inconsistency that occurs in its clinical presentation and progression. NPH can be similar, or occur in combination with, various diseases that are prevalent in the elderly, such as neurodegenerative disorders (e.g., senile dementia, Alzheimer’s and Parkinson’s), cerebrovascular disease, urological disorders, cervical or lumbar stenosis, and other diseases [7,8,9,10].

The European Multicenter Study on NPH reported a good correlation between a newly developed assessment scale and the clinical improvement, providing a promising tool for the diagnostic process [11,12]. Several studies reported different protocols for the diagnosis and management of NPH [13,14,15,16,17,18,19,20]. Moreover, different diagnostic tests, such as extended lumbar drainage (ELD), the intracranial pressure monitoring (ICPM), infusion test (IT) and tap test (TT), have been used to predict shunt responders [21,22,23,24]. However, additional clinical and radiological markers and prospective/randomized studies, along with CSF biomarkers, would improve the diagnostic process, especially the differential diagnosis with other neurodegenerative disorders, and could potentially identify the underpinning mechanisms of neurodegeneration.

This multicenter prospective observational study aims to evaluate the clinical outcomes of shunting in normal pressure hydrocephalus following a new uniform protocol.

## 2. Materials and Methods

### 2.1. Study Design and Guidelines

This is an observational study conducted at three institutions and approved by the IRB. The Strengthening the Reporting of Observational Studies in Epidemiology (STROBE) statement was the checklist for cohort studies that was used to define the study design.

### 2.2. Patients’ Population

Patients with NPH who underwent a ventriculoperitoneal shunt (VPS) between May 2015 and November 2019 at three institutions were prospectively considered for eligibility in the present investigation. A multidisciplinary team, including neurosurgeons, neurologists, neuropsychiatrists and neuroradiologists, validated these inclusion criteria: at least one of the symptoms of NPH triad (dementia, gait disturbance, and urinary incontinence); radiological signs on magnetic resonance imaging (MRI) of communicating hydrocephalus (Evans’ index, defined as the ratio of maximum width of the frontal horns of the lateral ventricles and the maximal internal diameter of the skull at the same level, ≥0.3); and the improvement of ≥5 points in the NPH scale, as described by Hallstrom et al. [25], after a one day external lumbar drainage (ELD) [26]. The stroke volume (SV), defined as the mean volume of CSF passing through the aqueduct during the measurement of both systole and diastole with a phase-contrast cine MRI, measures were collected preoperatively and during follow-up for all patients [27].

**Figure 1:**
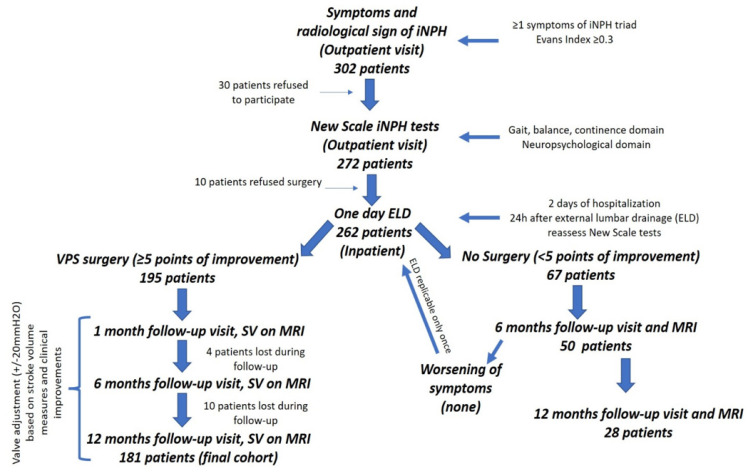
Flowchart of the study design.

The exclusion criteria were: other neurodegenerative disorders (excluding mild Alzheimer’s and Parkinson’s disease); secondary hydrocephalus; congenital and/or acquired neurological deficits; obstructive hydrocephalus; Karnofsky performance status (KPS) <60; and less than 12 months of follow-up. A flowchart of the study design is represented in Figure 1.

### 2.3. Clinical Outcomes

Demographical and clinical data were collected. The total score on the NPH scale, as described by Hallstrom et al., is the summed score of assessments in 4 domains, gait, neuropsychology, balance and continence, divided by 5 (gait has a double weight) or the number of available domain scores. All scores were converted to a min–max range from 0 to 100, where 100 is the performance of an age-matched healthy population. All domains and scores are summarized in Supplemental Material.

The different clinical domains were administered at one time by one neurologist in an outpatient visit, after the 1 day ELD test and at 1, 6 and 12 months after VPS surgery. Preoperative imaging, immediately a postoperative CT scan and MRI scan at 1, 6 and 12 months were also evaluated. 

### 2.4. Surgical Technique

All patients underwent a standard VPS procedure. In addition, before the insertion, multiple slits are created every 4 cm along the catheter in its intraperitoneal segment to create communications between the various compartments of the peritoneum crossed by the catheter. This makes a CSF distribution along the peritoneum compartments possible without accumulation in only one, avoiding pseudocyst formation [28]. The Codman—Hakim programmable (Integra) valve system and ProGav 2.0 (Aesculap—B. Braun) programmable valve system were implanted. Both valves had the SiphonGuard system (reduce the drainage rate when the flow dramatically increases during the transition from a horizontal to a vertical body position) incorporated and the first opening pressure was set at 110 mm H_2_O.

### 2.5. Statistical Analysis

A statistical comparison of the continuous variables was performed by Student’s *t*-test. A statistical comparison of the categorical variables was performed by the chi-square statistic using Fisher’s exact test (2-sided). The differences were considered significant at *p* < 0.05. Statistical analyses were conducted using StatView version 5 software (SAS Institute Inc., Cary, NC, USA).

## 3. Results

### 3.1. Demographical and Surgical Data

A total of 195 patients underwent VPS surgery for NPH at our neurosurgical departments in the investigation period: 14 (7.2%) patients were excluded because they were lost during follow-up for different reasons, and 181 (92.8%) patients matched the inclusion criteria and were finally included in the present investigation. The mean age of the included patients was 73.6 years ± 6.7 (range 59–86), and there were 97 (53.6%) women and 84 (46.4%) men. The mean follow-up was 38.3 months ± 17.3 (range 13–67). The most common symptoms reported were gait disturbance in 173 patients (95.6%), cognitive impairment in 158 (87.3%) and urinary incontinence in 130 (71.8%). The triad of symptoms was present in 128 patients (70.7%). The median preoperative length of symptoms was 21 months (mean 21.3 ± 7.6).

The most common co-morbidity was cardiovascular diseases (60.2%), followed by diabetes mellitus (31.5%), obesity (24.9%) and respiratory disorders (21.5%). A total of 56 (30.9%) patients were smokers. A total of 44 patients were in ASA class II (24.3%), 126 (69.6%) in class III and 11 (6.1%) in class IV. The Codman–Hakim valve system was implanted in 49 (27.1%) patients, and the ProGav 2.0 in 132 (72.9%). The mean length of surgery was 44 min ± 11.4 (range 30–90 min), mean blood loss was 60 mL ± 16.5 (range 40–110), mean length of hospitalization was 2 (range 2–5) days, and every patient was mobilized within 24 h after surgery. No intraoperative complications were reported. The mean Evans’ index was 0.39 ± 0.11. The mean stroke volume was significantly reduced from 103.2 µL ± 25.4 preoperatively, to 94 µL ± 22.8 (*p* = 0.0003) postoperatively. The demographical and operative data are summarized in Table 1 and Table 2, respectively.

### 3.2. Clinical Outcomes

The mean gait domain improved from 58.5 (±14.3) to 70.1 (±13.4) at last FU (*p* < 0.001), the mean balance domain improved from 66.7 (±21.5) preoperatively to 72.4 (±19.2) at last FU (*p* = 0.001), and the mean continence domain improved from 69.9 (±20.5) preoperatively to 76 (±22) at last FU (*p* = 0.002). The neuropsychological domain improved from 57 (±12) to 60.2 (±13) (*p* < 0.011). The mean functional scale score changed from 57.2 (±7.4) to 70.4 (±5.9) at last FU (*p* < 0.05), and the mean symptomatic scale score improved from 34.5 (±6.8) to 17.7 (±4.5) (*p* < 0.05). The overall improvement after 12 months was present in 91.2% (165) of patients. Five patients died during follow-up for other reasons not related to NPH or surgery. The clinical outcomes are summarized in Table 3. 

### 3.3. Valve Pressure Setting

Changes in the valve pressure were performed for 169 patients (93.4%) during follow-up. The first opening pressure was 110 mm H_2_O in all patients. The choice of changes of the opening pressure (± 20 mm H_2_O) was decided based on clinical symptoms and changes of the stroke volume on MRI. The new lower pressure was set to enhance the clinical improvement in cases with worsening symptoms and in case of increasing stroke volume measures on follow-up MRIs.

### 3.4. Complications and Reoperation Rate

A total of 16 complications were recorded (overall complication rate of 8.8%): 8 patients (4.9%) had a subdural hematoma due to over-drainage that spontaneously recovered after setting the opening pressure at 180 mm H_2_O; 2 patients (1.1%) had a postoperative intraparenchymal hemorrhage that spontaneously recovered within 3 weeks; 2 patients (1.12%) had a superficial wound infection medically treated with oral antibiotic therapy and recovered in 2 weeks; and 4 patients (2.2%) had a postoperative incisional hernia that required a revision surgery.

A total of 17 patients (9.4%) required surgical revisions for shunt malfunction; 3 patients (3 of 17; 17.6%) presented with proximal catheter obstruction; 7 patients (7 of 17; 41.2%) presented with a non-functioning valve system; and 7 patients (7 of 17; 41.2%) presented with peritoneal catheter extrusion. The complication and reoperation rates are summarized in Table 4.

### 3.5. Subgroup Analysis

Subgroup analysis was conducted for investigating how single factors may independently influence clinical outcomes. In the univariate analysis, the Evans’ index (*p* = 0.01), stroke volume (*p* = 0.024), and length of preoperative symptoms before surgery (*p* < 0.001) independently influenced the clinical outcomes, providing lower chances of responsiveness to surgery. However, a multivariate analysis (Table 5) showed that only the stroke volume (*p* = 0.0001) and duration of the preoperative symptoms (*p* = 0.037) should be considered as independent factors for non-responsiveness to shunting in this cohort of patients.

## 4. Discussion

This multicenter prospective observational study on the clinical outcomes of shunting in NPH patients resulted in the following clinically relevant conclusions: (1) a multidisciplinary team focused on this disease and a uniform protocol is needed to accurately select patients and achieve a correct diagnosis of NPH; (2) surgery with VPS improves gait, balance and continence domain and some of the neuropsychological domain with an impact on quality of life and daily activities of patients; (3) the efficacy of shunting occurred within 3 months after surgery and persisted with a slight reduction at 12 months follow-up in 90% of patients; (4) the one-day ELD showed a high sensibility to select patients with NPH; (5) the use of programmable valves with the anti-siphon system reduce the complication rate related to over drainage; and (6) clinical improvement and the stroke volume measures are necessary to determinate the valve adjustment during follow-up and for the management of NPH in a long-term period.

In our study, as previously reported in other studies [16,17,18,19,20], the gait and balance domain demonstrated the highest mean improvement rates. In contrast with previous studies [16,17,19], we noted a higher improvement for urinary incontinence and the neuropsychological domain. However, during follow-up, the cognitive functions continue to deteriorate, especially in patients with other mild neurodegenerative disorders. Furthermore, controversy still exists on the reversibility of cognitive impairment, and several studies demonstrated that the gait domain has the highest improvement and impacts the total score the most [11,14,16].

After 1 year, 91.2% of patients had an improvement of clinical outcomes after shunting and our results are consistent with the previous studies reporting the clinical benefit of shunt, ranging from 31% to 96% [18,19]. Toma et al. [20], in their systematic review, reported that results from studies published in the last 5 years showed 82% improvement following shunt insertion, a mortality of 0.2%, and a combined common complications rate of 8.2%. A recent systematic review by Giordan et al. [29], which compared the outcomes of different surgical techniques of 33 studies with 2461 patients, reported an improvement in functional performance in more than 75% of patients without significant differences among the different techniques utilized. 

In our study, the length of the preoperative symptoms was a significant outcome predictor, as reported in other investigations [30,31]. A recent retrospective study of 393 patients treated with shunting reported that a longer symptom length was significantly associated with worse outcomes. However, no statistically significant differences were observed in these outcomes at the last follow-up (median: 31 months) [31].

Of interest, in responders to VPS, we found a postoperative reduction in stroke-volume values on MRI. In our opinion, stroke volume should be considered not only as an outcome predictor, but is also crucial during follow-up for valve adjustments [32]. To the best of our knowledge, this study provides an analysis of the effects of stroke volume on the outcomes and postoperative pressure settings of the programmable valves. Furthermore, the SiphonGuard system used for all patients reduced the complication rate related to over-drainage (4.4% of patients in our study, which resolved after increasing the opening pressure), in keeping with other investigations [33,34]. An over-drainage complication rate after the treatment of NPH is reported (about 10% and 85%), which is spontaneously resolved after increasing the opening pressure [35,36]. The overall complication rate (8.8%) and reoperation rate (9.4%) in this cohort are similar to those reported in the literature and commonly observed with programmable valves [37,38,39].

The 91.2% response rate at the last follow-up (mean: 38 months) in the present investigation is attributed to our selection criteria, which include clinical, radiological, and the one-day ELD test. In our opinion, the one-day ELD and the stroke volume measures preoperatively and during follow-up are the key factors that correctly identify the shunt responders’ patients. The one-day ELD test is a reliable tool in NPH diagnosis and predicting patients that will have an improvement in clinical outcomes after shunting surgery, with a sensitivity, specificity and accuracy of 100%, 75.0% and 97.1%, respectively [27]. However, other studies use the continuous ICP monitoring as a diagnostic tool with similar accuracy [23,24]. A recent systematic review showed that ICPM is statistically the most effective diagnostic test, followed by ELD [21]. In contrast, Mahr et al. showed that a response to ELD yielded the best prediction for the improvement of symptoms following surgery [22]. These results show that, nowadays, uncertainty still remains concerning which diagnostic test to choose. Of interest, in the non-responders to ELD, ICP monitoring could be used to assess false negatives.

The stroke volume, measured on phase-contrast cine MRI, seems to increase between the onset of the symptoms and the following 18 months, then there is a plateau phase for another 18–20 months, and finally it decreased. However, after its decrease, the patient’s clinical symptoms progressively deteriorate. The progressive reduction in the stroke volume in untreated patients with deteriorating clinical symptoms may be a sign of a progressive cerebral ischemic injury, which renders the NPH symptoms irreversible [32,40]. The SV decrease after shunting and an increase in the clinical deterioration during follow-up highlight that the open pressure is not adequate and should be adjusted to a lower pressure. Morover, in case of valve malfunctions, the SV continues to increase after adjustment. At last, a relevant decrease in the SV values could indicate over-drainage and could prevent related complications. However, its implication should be investigated in further studies with larger cohorts and a longer follow-up.

Certainly, the goal of diagnosis is to appropriately refer patients with NPH for shunt surgery and to correctly identify those who do not have NPH or will be non-responders, and suggest against shunting, thus accordingly minimizing the undiagnosed NPH patients or the risks of unnecessary surgery.

### Limitations of This Study

There are some limitations to be disclosed. Firstly, the patient cohort is small and follow-up is not too long, and certain complications and outcomes could have been consequently unrecognized. Secondly, our results should be validated in other cohorts using similar clinical and radiological measures, and comparative studies of different diagnostic tools are necessary. Finally, this study does not have a control group, which results in lower levels of evidence. Further randomized studies need to be performed with a larger cohort and longer follow-up to validate our findings.

## 5. Conclusions

Surgical treatment by VPS for NPH improves symptoms in most patients, when accurately selected. Shunting has a low complication rate and guarantees clinical improvements postoperatively for >90% of patients. Appropriate diagnostic procedures and clinical tests that result in accurate patient selection, are the key factors for an acceptable outcome after VPS. At last, careful clinical follow-up and stroke volume measurement on MRI are necessary for valve adjustments to optimize the long-term success of shunt therapy in NPH. Further randomized and comparative clinical trials would better clarify the role of diagnostic procedures on early diagnosis and its theorical impact on long-term clinical outcomes.

## Figures and Tables

**Table 1:** Patient characteristics.

**Total Number of Patients**	**181**
**Mean age, years ± SD (range)** **Mean follow-up, months ± SD (range)**	73.6 ± 6.7 (59–86) 38.3 ± 17.3 (13–67)
**Sex**	
Female Male	97 (53.6%) 84 (46.4%)
**ASA classification**	
I II III IV V	0 44 (24.3%) 126 (69.6%) 11 (6.1%) 0
**Symptoms**	
Gait disturbance Cognitive impairment Urinary incontinence Complete triad	173 (95.6%) 158 (87.3%) 130 (71.8%) 128 (70.7%)
**Preoperative duration of symptoms (median)**	21 months
**Comorbidity**	
Cardiovascular diseases Diabetes mellitus Obesity Respiratory diseases Smokers	109 (60.2%) 57 (31.5%) 45 (24.9%) 39 (21.5%) 56 (30.9%)

**Table 2:** Operative characteristics.

	MEAN ± SD
**Radiological signs on MRI**	
Evans’ Index Stroke Volume	0.39 ± 0.11 103.2 ± 25.4
	**Nr. (%)**
**Type of shunt system**	
Codman–Hakim (Integra) Pro-Gav 2.0 (B. Braun)	49 (27.1%) 132 (72.9%)
	**Nr. (range)**
**Mean length of surgery ± SD (range)**	44 min ± 11.4 (30–90 min)
**Mean length of hospital stay (range)**	2 days (2–5 days)
**Mean time of postoperative mobilization**	1 day (1–3 days)
**Intraoperative blood loss ± SD (range)**	60 mL ± 16.5 (40–110 mL)

**Table 3:** Clinical outcomes.

	MEAN ± SD
**Gait domain**	
Preoperative	58.5 ± 14.3
Postoperative	66.0 ± 12.2
Follow-up at 12 months	70.1 ± 13.4
*p*-value (pre vs. follow-up)	**<0.001**
**Balance domain**	
Preoperative	66.7 ± 21.5
Postoperative	72.4 ± 19.2
Follow-up at 12 months	71.7 ± 22.1
*p*-value (pre vs. follow-up)	**0.001**
**Neuropsychological domain**	
Preoperative	57.0 ± 12.0
Postoperative	62.0 ± 10.3
Follow-up at 12 months	60.2 ± 13.0
*p*-value (pre vs. follow-up)	**0.011**
**Continence domain**	
Preoperative	69.9 ± 20.5
Postoperative	78.3 ± 18.2
Follow-up at 12 months	76.0 ± 20.0
*p*-value (pre vs. follow-up)	**0.002**

**Table 4:** Complication and reoperation rates.

	Nr. (%)
**Complications**	
Subdural hematoma/hygroma	8 (4.4%)
Ischemic/hemorrhage	2 (1.1%)
Infection	2 (1.1%)
Incisional hernia	4 (2.2%)
Overall complication rate	**8.8%**
**Reoperation rate**	
Shunt malfunctioning Incisional hernia	17 (9.4%) 4 (2.2%)

**Table 5:** Univariate and multivariate analysis.

Risk Factors	Univariate Analysis			Multivariate Analysis *		
	OR	CI 95%	*p*-Value	OR	CI 95%	*p*-Value
Age	1.094	(1.034–1.157)	0.071			
Sex	0.896	(0.248–3.245)	0.817			
Stroke volume	1.405	(1.198–1.647)	<0.001	1.463	(1.227–1.719)	0.0001
Evans index	1.143	(1.037–1.219	0.01			
Preoperative symptoms	1.452	(1.271–1.741)	<0.001	1.265	(1.028–1.557)	0.037

* Backward stepwise (conditional) method in binary logistic regression analysis; the non-significant factors are eliminated during analysis.

## Data Availability

Not applicable.

## Supplementary Materials

The following supporting information can be downloaded at: https://www.mdpi.com/article/10.3390/jcm11051286/s1, Supplemental Material on the new scale in iNPH.
