# Supplementary material for: Clinical Outcomes of Shunting in Normal Pressure Hydrocephalus: A Multicenter Prospective Observational Study
Source: J Clin Med. 2022 Feb 26;11(5):1286. doi: 10.3390/jcm11051286 (PMC8911143; doi:10.3390/jcm11051286)
Supplement: Supplementary file 1 [file jcm-11-01286-s001.zip › jcm-1569503-supplementary.pdf]

Supplemental Material on the New Scale in iNPH

Gait domain

The gait domain is evaluated by measurements of the number of steps and seconds needed to walk 10 m at free pace, and an ordinal rating of the gait that is based on observations while the patient tries to walk heel-to-toe (tandem gait) and to turn 180° as followed:

1. Normal.
2. Slight disturbance of tandem walk and turning.
3. Wide-based gait with sway, without foot corrections.
4. Tendency to fall, with foot corrections.
5. Walking with cane.
6. Bi-manual support needed.
7. Aided.
8. Wheelchair bound

The gait domain score is the mean of the three converted scores.

Balance domain

The balance domain is represented by observations of the patients' efforts to stand up straight on one or both legs as followed:

1. Able to stand independently for more than 30 s on either lower extremity alone.
2. Able to stand independently for <30 s on either lower extremity alone.
3. Able to stand independently with the feet together (at the heels) for more than 30 s.
4. Able to stand independently with the feet together for <30 s.

5. Able to stand independently with the feet apart (one-foot length) for more than 30s.
6. Able to stand independently with the feet apart for <30 s.
7. Unable to stand without assistance.

The rating is converted into scores as followed: 1 = 100, 2 = 83, 3 = 67, 4 = 50, 5 = 33, 6 = 17 and 7 = 0.

Continence domain

Continence is represented in this scale as follows:

1. Normal.
2. Urgency without incontinence.
3. Infrequent incontinence without napkin
4. Frequent incontinence with napkin
5. Bladder incontinence
6. Bladder and bowel incontinence.

The results are converted into scores as followed: 1 = 100, 2 = 80, 3 = 60, 4 = 40, 5 = 20 and 6 = 0.

Neuropsychological domain

The following tests were administered at one time and by one neuropsychologist in an outpatient visit; immediately after the one-day ELD test and at one, 6- and 12-months after VPS surgery:

1. **Grooved Pegboard** (Lafayette Instrument Co.), measuring manual dexterity as time to fit 25 pegs into holes with randomly positioned slots measured as time in seconds for the Dominant and Non-dominant hand and the Sum of both;

2. The Rey Auditory Verbal Learning Test (RAVLT), measuring verbal learning and memory measured as the total number of recalled words over five learning trials (Learning) and the number of recalled words after 30 min of distraction (Delayed Recall);

3. The Stroop test, measuring Color Naming speed and Response Selection which requires the patient to name the colors (blue, red, green or yellow) of 100 rectangles as fast as possible, and then to name the printed color of 100 incongruent color words (e.g., the word blue printed in red). The scores are the number of seconds for each trial (Color Naming and Response Selection, respectively) and the increase in time from the first to the second trial.

4. Digit Span, measuring working verbal memory. The examiner reads a list of numbers as the digit sequencing, and the participant repeats them until an incorrect answer is given. It can be modulated to digit spans of 1-100 sequences long, with forward and reverse sequencing available as features.

5. **Trial Making Test**, measuring visual attention and task switching. It consists of two parts in which the subject is instructed to connect a set of 25 dots as quickly as possible while still maintaining accuracy
